# Assessment of the Simultaneous Use of Biomaterials in Transalveolar Sinus Floor Elevation: Prospective Randomized Clinical Trial in Humans

**DOI:** 10.3390/ijerph17061888

**Published:** 2020-03-14

**Authors:** Adrián Maximiano Millán, Rocío Bravo Álvarez, Miguel Plana Montori, María Guerrero González, David Saura García-Martín, Blanca Ríos-Carrasco, Francesca Monticelli, José Vicente Ríos-Santos, Ana Fernández-Palacín

**Affiliations:** 1 Periodontics, Faculty of Health and Sport Sciences, Universidad de Zaragoza, C/Velódromo S/N, 22006 Huesca, Spain; adrian.maximiano@hotmail.com (A.M.M.); rociobravo24@hotmail.com (R.B.Á.); miguelplanamontori@gmail.com (M.P.M.); drmguerrero@gmail.com (M.G.G.); clinicasaura@gmail.com (D.S.G.-M.); fmontice@unizar.es (F.M.); 2 Advanced Periodontics, School of Dentistry, Universidad de Sevilla, C/Avicena S/N, 41009 Sevilla, Spain; brios@us.es; 3 Department. of Sociosanitary Sciences Facultad de Medicina, Universidad de Sevilla, Avda. Dr. Fedriani, S/N, 41009 Sevilla, Spain; afp@us.es

**Keywords:** osteotome, transalveolar sinus floor elevation, bone grafting

## Abstract

Implants inserted in the posterior maxilla frequently need additional surgery for successful bone augmentation. One of the most common procedures for this is transalveolar sinus floor elevation. There are different protocols for this procedure, and there is controversy over the simultaneous application of grafting material upon elevating. In this prospective randomized clinical study in humans, a total of 49 transalveolar sinus floor elevations were performed in 49 different patients, divided into a control group (without graft, 25 patients) and a test group (with graft, 24 patients). The analyzed variables were obtained through digital orthopantomography on day 0 (day of surgery) and 18 months after surgery. These measurements showed a tendency towards greater vertical bone gain in the test group, but this was not statistically significant. Therefore, considering that sinus elevation and implant placement without the application of grafts is a successful treatment with fewer complications, a critical assessment of the need for these biomaterials is necessary.

## 1. Introduction

Since the 1970s, classical longitudinal studies have shown that the first teeth to be lost due to periodontal disease are the maxillary molars, whose prolonged absence causes a reduced bone volume due to pneumatization of the maxillary sinus and alveolar bone resorption from the lack of mechanical stimulation [1,2,3,4,5,6,7]. The success of implant therapy is directly related to primary stability, and in turn, to the bone volume present at the implant site. As a result, the long-term prognosis can be poor due to the presence of insufficient bone volume [8,9,10,11,12].

The first author to propose a transalveolar approach to correct the pneumatization of the sinus cavity and place implants was Tatum in 1986 through a “socket former”, which is the same size as the implant to be placed [13,14]. In 1994, Summers proposed the use of tapered osteotomes with increasing diameters to conserve more bone when drilling was not carried out. After elevating, autografts, allografts, xenografts, or synthetic materials were added [15,16,17,18].

Currently, the simultaneous use of grafts for sinus floor elevation in the transalveolar approach is a subject of controversy. Many authors consider the ‘tent’ effect produced after fracture and displacement of the cortical bone at the sinus floor allows the formation and stabilization of blood clots and spontaneous bone formation, this minimizes the risks of introducing material into the sinus [19,20,21]. The objective of this article is to evaluate whether the use of biomaterials together with the elevation of the transalveolar sinus floor is justified. This way done by comparing the radiographic vertical bone gain at 18 months and evaluating the covering at the region apical to the implant.

## 2. Results

**Figure 1:**
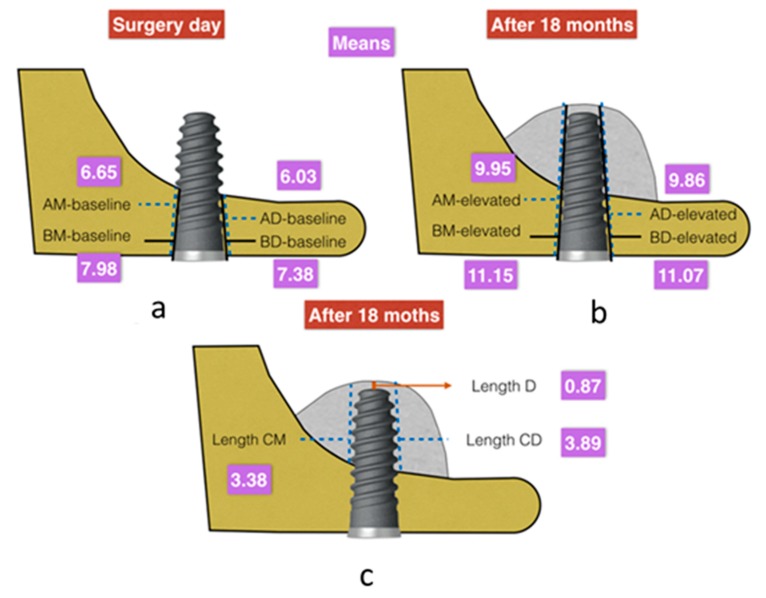
(**a**) Mean values obtained for the baseline variables analyzed in the radiographic measurements on the day of surgery (expressed in millimeters). (**b**,**c**) Mean values obtained (mm) for the elevation and intra-sinus bone gain variables analyzed in the radiographic measurements after 18 months of surgery (expressed in millimeters).

There were not significative differences in baseline values in both groups. Any baseline differences were less than 0.54 mm in all variables analyzed and without statistical significance (Figure 1).

**Figure 2:**
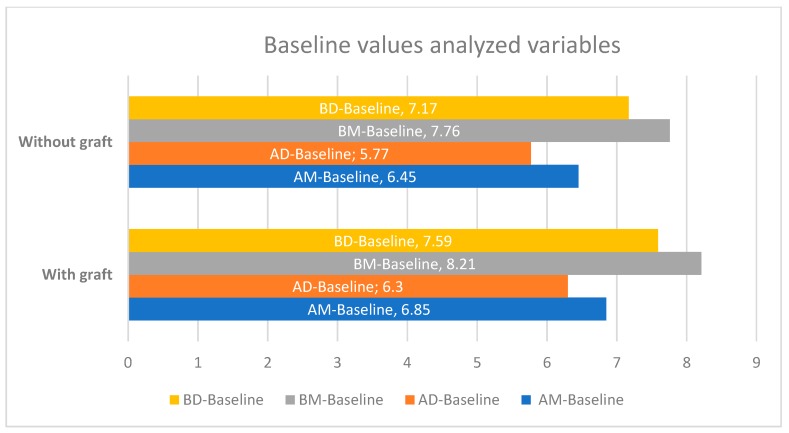
Differences in baseline values between the test and control groups (expressed in millimeters).

In this study, minimum baseline cortical values (AM-baseline) of 6.65 ± 1.32 mm (95% CI −0.36, 1.16, *p* = 0.299) mesially and 6.03 ± 1.42 mm distally (AD-baseline) (95% CI −0.3,1.23, *p* = 0.263) were obtained (Table 1 and Figure 2). There were no statistically significant differences in variables between the test and control groups. There were also no differences if the implant shoulder was taken as a reference mesially (BM-baseline) (7.98 mm ± 1.35, 95% CI −0.3,1.23, *p* = 0.263) or distally (BD-baseline) (7.37 mm ± 1.41, 95% CI −0.39,1.24, *p* = 0.292).

These values after the transalveolar elevation technique (global means) were 9.94 mm (±1.18) for AM-elevated and 9.86 mm (±1.24) for AD-elevated (Figure 2). Taking the implant shoulder as a reference, these measurements were 11.14 mm (±1.14) for BM-elevated and 11.07 mm (±1.12) for BD-elevated.

The mean distance from the apex of the implant to the new base of the maxillary sinus (length D) was 0.87 mm (±0.47), with a maximum value of 1.88 mm (Figure 2). The ‘tent’ was elevated mesially by 3.37 mm (±1.15) and distally by 3.89 mm (±1.11).

**Figure 3:**
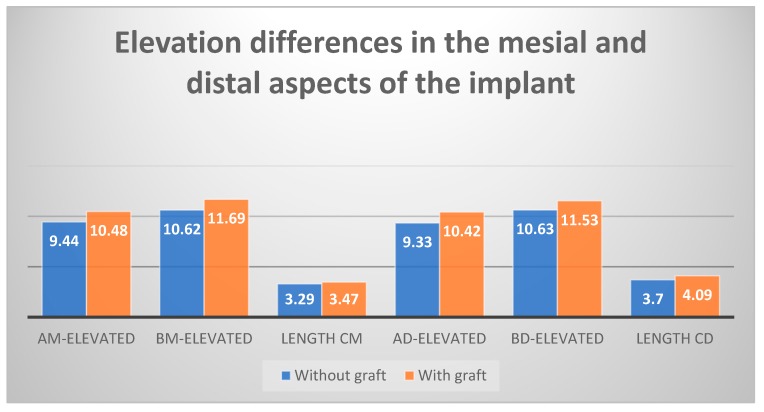
Differences in elevation between the mesial and distal aspects of the implant after 18 months, test group vs. control group (expressed in millimeters).

**Figure 4:**
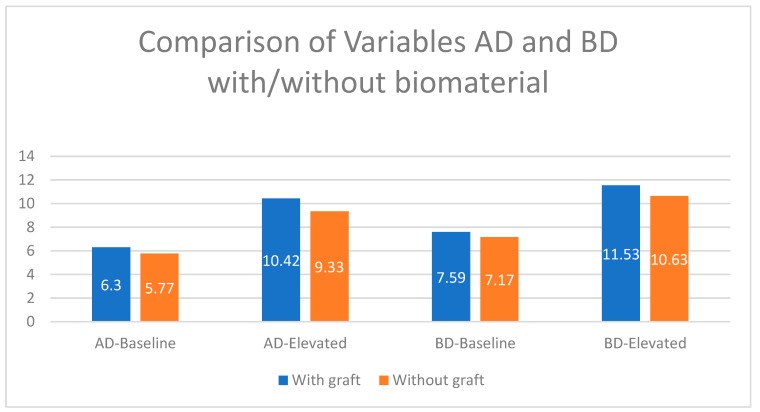
Dimensional changes in the analyzed variables compared to baseline, test group vs. control group (expressed in millimeters).

**Figure 5:**
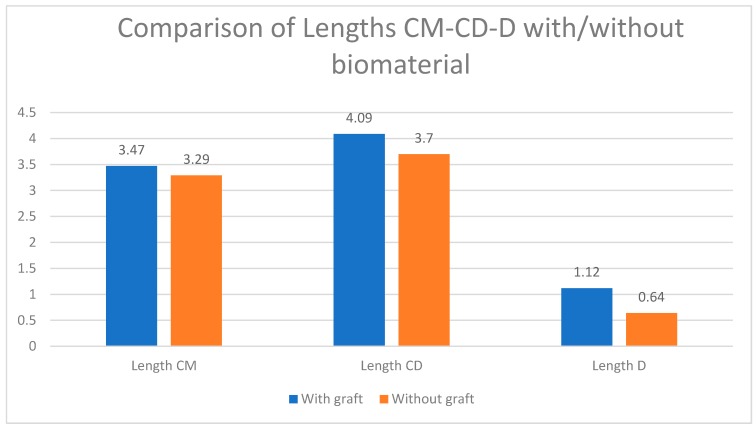
Dimensional changes in the variables of elevation and intra-sinus bone gain, test group vs. control group (expressed in millimeters).

When analysis was carried out by the study group, we found that there was a greater difference between baseline and elevated values in the test group (with biomaterial) compared to the control group and that this difference was accentuated in the distal aspect of the implant (Figure 3). Looking only at the elevated values, this difference was repeated when length CD (the distance from the initial sinus baseline to the new sinus baseline in the distal aspect of the implant), length CM (the distance from the initial sinus baseline to the new sinus baseline in the mesial aspect of the implant), and length D were analyzed. We always saw larger values in the test group, with CD and D being particularly large, but only the latter was statistically significant (Figure 4 and Figure 5). When analyzing the magnitude of elevation obtained in both groups (subtraction of elevated values from baseline values), it was evident that in all the analyzed lengths, both mesially (AM-elevated) and distally (AD-elevated), better results were achieved in the test group (Table 1).

By individually analyzing all the elevated lengths, in radiographs analyzed 18 months after implant placement with elevation, we found that all variables had higher values in the group with biomaterial (Figure 2). These values observed were the highest distal to the implant. The BD-elevated length (10.63 mm in the control group and 11.53 mm in the test group) and the CD length (3.70 mm in the control group and 4.09 mm in the test) presented the greatest differences (Figure 4 and Figure 5). All the variables analyzed were normally distributed except for BM-baseline, AD-elevated, BD-elevated, and length D. Student’s t-test was used to compare the normally distributed variables, this showed statistical significance for all at 18 months (*p* < 0.005). The four non-normally distributed variables were compared by the Mann–Whitney U-test for independent samples, and only length D showed statistically significant differences (*p* < 0.005).

## 3. Discussion

In this study, all the radiographs are orthopantomographs done with the same orthopantomograph machine (KaVo Dental GmbH and Co. Biberach, Germany) with vertical magnification that was calibrated according to the length of the implant placed. For this reason, knowing the magnification average, we could adjust all measurements and calibrated mathematically.

In this study, we did not do horizontal measurements, which would have been an important limitation in the study due to orthopantomography distortion.

Summers transalveolar method proposed a crestal approach using tapered osteotomes of increasing diameters to conserve a larger amount of bone. Since drilling is not done, the adjacent bone is compressed by pushing and tapping as the sinus membrane is lifted. Next, autografts, xenografts, allografts, or synthetic materials are added to increase the volume below the elevated sinus membrane.

In our study, we obtained a 100% success rate and survival rate for all implants evaluated. Rosen et al. [15], in a multicenter study in which 173 implants that were submerged and placed by this technique were followed up. These implants showed a success rate of 96% at 18 months of loading [13]. It is thought that this is due to the elevation of the sinus floor with osteotomes combined with bone grafts (i.e., bone-added osteotome sinus floor elevation) and so is more conservative and less invasive [13].

Although the results obtained in this study showed a success rate of 100% for both study groups and no complications were reported, it is important to note that some authors, such as Nedir et al. [22,23,24], in their prospective, randomized, controlled studies, saw a higher complication and a success rate at 90% in the grafted group compared to 94.1% in the control group; this is after 5 years of evaluation. Any differences in success rates compared to our findings could be due to one of their inclusion criteria being the residual bone height of ≤4 mm, and while this study obtained baseline means of 6.65 mm mesially and 6.03 mm distally with the minimum baseline values of 4.17 mm mesially and 3.56 mm distally. The current scientific literature indicates that, taking into account that bone grafts are placed blindly in the space below the sinus membrane, there is an increased risk of complications when the residual alveolar bone height is less than 4 mm.

In our study, the maintenance of the patient in periodontal health was very important, as we know from the studies carried out by Patini et al. and Martellacci et al. [25,26] that the oral microbiota can negatively influence the success rates of our implant therapy.

There is controversy about the need to place graft material to maintain space for the formation of new bone after elevating the sinus membrane using the osteotome technique. Regardless of the measurement used, we found no inter-group differences in vertical bone gain in either the mesial or distal aspect of the implants. These results agree with those obtained by Markovic et al. [27] in their prospective randomized study of a total of 200 implants. They found that all implants achieved intra-sinus bone filling, which was significantly higher in the graft group in initial measurements. However, these values were the same after 29.7 months due to the volumetric loss detected in both groups. Therefore, it can be concluded that the use of biomaterials does not influence implant survival, nor is it a requirement for future osteogenesis.

Along the same lines, Yang et al. [28] performed a retrospective study on 51 implants by analyzing the radiographic changes found in the first months. They used implants of similar diameter to ours (4.5–5 mm) and with lengths of 6–8 mm. The mean intra-sinus new bone formation joining the mesial and distal aspects in our study was 3.49 mm in the control group and 3.78 mm in the test group. The values of the test group resemble those obtained by Yang et al. [28] (mean of 3.96 ± 2.38), but their values in the control group were lower (mean of 1.29 ± 1.07 mm). The authors conclude that there were no statistically significant differences between the mean length of implant protrusion into the sinus in the ungrafted group vs. the grafted group after elevation. A recent meta-analysis and systematic review of the literature by Chen and Shi [29] obtained significant differences in intra-sinus bone gain between the grafted group (4.1 mm) and the control group (2.9 mm). Their study indicates that there were no statistically significant differences, but some authors, such as Pjetursson, suggest that there is a greater probability of bone neoformation when grafts are used, and others, such as with Nedir et al. [22,23,24], emphasize that in 100% of graft cases, neoformation is always greater than 2 mm, compared to 93.8% of cases in the control group.

A recent systematic review and meta-analysis conducted by Aludden et al. [30] showed mean intra-sinus bone gain similar to those in our study. In the non-graft group, it was 3.2 mm, while in the group grafted with Bio-Oss® (Geistlich, Switzerland), it was higher. These differences disappeared on radiography after the first year due to graft contraction. Caban et al. [31] and Nedir et al. [32], both in studies with a single group without graft material and with 68 and 25 implants, respectively, used radiographic analysis to demonstrate that the use of a graft was not necessary to obtain intra-sinus bone gains, as the groups had average gains of 3.2 mm (with a maximum value of 4.6 mm) and 3.14 mm, respectively. These results are in line with ours (mean gain without grafting of 4.52, maximum of 4.98 mm).

Although the variables analyzed in this study do not seem to clarify the controversy present in the current literature, the variables analyzed show a slight tendency to be higher in the test group than in the control group. This may be because, when introducing biomaterials, the Schneider membrane moves further away from the apex of the implant. For this reason, length D was the only variable to show statistically significant differences. This variable has not been analyzed by other authors, who consider the protrusion of an implant into the sinus as a “zero” space between the apex of the implant and Schneider membrane.

These slight tendencies are always greater in the measurements performed on the distal aspects of the placed implants. This may be due to the shape of the sinus (always being more pneumatized distally), the effect of gravity when blindly introducing the biomaterial (as in sinus lifts with a lateral window approach), or the residual bony plate being irregular and almost always less in the distal zone. In this case, since it is a tissue-level implant, and aiming to not leave a treated surface exposed distally, sometimes the implant is submerged more mesially, causing crestal bone remodeling and decreasing the dimensions achieved mesial to the implant. The authors mentioned above did not analyze the possible differences between the mesial and distal aspects of the placed implants.

In the discussion about the degree with we can generalize to the rest of the population the results of this study, we could highlight that the risk of bias in the selection was eliminated (balanced randomization has already been discussed, as well as the homogeneity of onset in the residual flanges of both groups). On the other hand, there is no Hawthorne effect in this technique whereby the subject, knowing that it is measured, can influence the results.

## 4. Materials and Methods

### 4.1. Study Population and Inclusion Criteria

The study was conducted at the School of Health and Sports Sciences of the University of Zaragoza at the Dental Practice Center. The ethics committee of the University of Zaragoza (CEIC Aragon) approved this study, as reflected in its Act No. 04/2019.

Patients older than 18 years, with good general health status (American Society of Anesthesiologists status I or II), who presented partial posterior maxillary edentulism, and who wanted rehabilitation through dental implants were included. Patients had to have adequate bone thickness for implant placement (residual bone height of at least 5 mm), and, at the discretion of the physician would require sinus elevation with osteotomes. All patients were included in a periodontal program, and if required, were operated on without signs of periodontal disease (when we find bleeding of probing and probing depth greater or equal than 4 mm.). In this study, implants were only included that were to be placed in areas where at least 4 months had elapsed after dental extractions and in areas that had not undergone previous bone regeneration.

The sample size was calculated with the program N Query Advisor, this was based on the study of Nadir, with 3.9 mm on average for the test group and 5.0 mm for the control group with a common standard deviation of 1.0 and a statistical power of 80% in a two-tailed study with statistical significance set at *p* < 0.05 [22]. According to the established inclusion criteria, a total of 49 patients were selected (*n* = 49), they were randomly distributed into two groups:

- Control (implant placement without graft material), *n* = 25;
- Test (implant placement with graft material), *n* = 24.

Patients were assigned to the groups randomly. A balanced randomization with 50 letters was done: 25 controls and 25 tests (we lost a patient because he changed his location). With this method, we guarantee the homogeneity of both groups, that probably, we could not have reached throwing a coin.

The calibrated researcher that made the measurements was blind, ignoring the membership of each group.

### 4.2. Surgical Procedure

The surgery was performed under aseptic conditions by preparing a sterile surgical field in the dental operating room. Antibiotic prophylaxis was administered, consisting of 2 g amoxicillin or 600 mg of clindamycin + 500 metronidazole in cases of allergies, 1 h before surgery. Pre-surgical patient preparation included oral rinsing with 0.1% chlorhexidine for one minute, followed by buccal and palatal administration of 40.0 mg articaine hydrochloride with 40/0.005 mg/mL epinephrine for local anesthesia.

A crestal incision was made with or without releasing, and a full mucoperiosteal flap was lifted. The optimal three-dimensional positions were chosen via a surgical stent, and drilling was performed with the pilot drill up to 1.5–2 mm from the sinus floor. Then, the elevation was performed with the first osteotome, creating a “greenstick” fracture of the sinus floor, progressively increasing the diameter of each osteotome used until it reached approximately 1.5 mm smaller than the implant diameter to be placed.

Once elevation was complete, randomized patients received the implant (control group) or were treated with approximately 0.2–0.3 g of graft material (Bio-Oss®, Geistlich, Switzerland) before implant placement (experimental group). Previously, clinical verification of the integrity of the Schneiderian membrane (Valsalva manoeuvre) was performed.

All patients were treated with Klockner Essential Cone® implants (SOADCO S.L., Andorra) with a standard platform of 4.5 mm, varying the implant diameter between 3.5 and 4.5 mm, depending on the horizontal available bone (width of the existing ridge), maintaining at least 1.5 mm of bone around the implant site. All patients received the same postoperative advice, and after surgery, antibiotic treatment (amoxicillin 750 mg/8 h or clindamycin 300 mg/8 h + metronidazole 250 mg/8 h) and anti-inflammatory treatment (ibuprofen 600 mg/8 h) was prescribed. Gastric protection consisted of omeprazole capsules 20 mg/24 h.

All patients underwent digital orthopantomography on the day of implant placement.

### 4.3. Statistical Analysis

The variables analyzed were as follows:

- Initial available bone: Amount of bone measured on a calibrated panoramic radiograph.
- Implant length: The length of the implant used was 8 or 10 mm.
- Postoperative radiography:

Postoperative orthopantomography was done to assess the correct placement of the implant relative to the adjacent structures and to allow different measurements.

The measurements collected on the day of surgery were as follows:

- Length AM-baseline: Distance between the alveolar process and cortical bone of the sinus floor, mesial to the implant.
- Length BM-baseline: Distance between the implant shoulder and the cortical bone of the sinus floor, mesial to the implant.
- Length AD-baseline: Distance between the alveolar process and the cortical bone of the sinus floor, distal to the implant.
- Length BD-baseline: Distance between the implant shoulder and the cortical bone of the sinus floor, distal to the implant.

Measurements recorded at 18 months were:

- Length AM-elevated: Distance between the alveolar process and the new sinus cortical bone, mesial to the implant.
- Length BM-elevated: Distance between the implant shoulder and the new sinus cortical bone, mesial to the implant.
- Length AD-elevated: Distance between the alveolar process and the new sinus cortical bone, distal to the implant.
- Length BD-elevated: Distance between the implant shoulder and the new sinus cortical bone, distal to the implant.
- Length CM: Distance between the initial sinus cortical bone and the new cortical bone, mesial to the implant.
- Length CD: Distance between the initial sinus cortical bone and the new cortical bone, distal to the implant.
- Length D: Distance from the apex of the implant to the new cortical bone.

(Figure 1a–c).

Given that the orthopantomographs can distort the image (i.e., magnify it), the implants were measured, and the degree of distortion for each variable was calculated before the analysis. Each variable was measured three times (24 h apart) by the same operator calibrated with a previous kappa of 0.99. The intraclass correlation coefficient of the three measurements showed a very good correlation, so the mean of the three measurements was taken for analysis.

Statistical analysis was performed using IBM SPSS Statistics 24 software (IBM, New York, EEUU). First, statistical data cleansing (exploration) was performed by numerical methods and graphs. The quantitative variables were summarized as the means and standard deviations. Descriptive analyses were completed with the corresponding graphical representations. The qualitative variables were expressed as percentages. Inter-group comparisons were made with Student’s t-test (after checking for normality with the Shapiro–Wilk test). Those variables that were not normal were compared with the Mann–Whitney U-test for independent samples. A significance level of *p* < 0.05 and a confidence interval of 95% was established.

## 5. Conclusions

Transalveolar sinus elevation is a predictable treatment with or without biomaterials. Although the values achieved were higher with the use of a biomaterial, there was no statistical or clinical differences that makes it necessary for transalveolar elevation. Although there were no postsurgical complications in this study, the use of biomaterials in transalveolar elevations may pose an increased risk of complications for the patient. Further studies are needed with a longer follow-up and a similar methodology to verify whether the use of biomaterials could provide better support to the clinical results achieved.

## Figures and Tables

**Table 1:** Bone gain in mm mesially and distally from the alveolar crest border to the sinus floor (intercortical) and from the implant neck to the sinus floor. Height of floor elevated mesially, distally, and apically to the implant.

DISTANCES AT CRESTA ALVEOLAR LEVEL	Gain (mm)			
	Without Biomaterial	With Biomaterial	*p*	Confidence Interval 95%
intercortical bone, mesial	2.98	3.62	0.001	0.42/1.66
intercortical bone, distal	3.56	4.12	0.005	0.39/1.83
from implant neck to floor, mesial	2.86	3.48	0.001	0.48/1.65
from implant neck to floor, distal soil	3.47	3.94	0.006	0.66/1.54
floor elevation, mesial	3.28	3.47	0.591 *	−0.49/0.85
floor elevation, distal	3.70	4.09	0.225 *	−0.25/1.02
bone above implant	0.63	1.12	<0.0005	0.27/0.77

* No statistical significance.
